# Vehicle Counting Based on Vehicle Detection and Tracking from Aerial Videos

**DOI:** 10.3390/s18082560

**Published:** 2018-08-04

**Authors:** Xuezhi Xiang, Mingliang Zhai, Ning Lv, Abdulmotaleb El Saddik

**Affiliations:** 1 The School of Information and Communication Engineering, Harbin Engineering University, Harbin 150001, China; zmlshiwo@outlook.com (M.Z.); lvning@hrbeu.edu.cn (N.L.); 2 The school of Electrical Engineering and Computer Science, University of Ottawa, Ottawa, ON K1N 6N5, Canada; elsaddik@uottawa.ca

**Keywords:** vehicle counting, unmanned aerial vehicle, vehicle detection, visual tracking, aerial video

## Abstract

Vehicle counting from an unmanned aerial vehicle (UAV) is becoming a popular research topic in traffic monitoring. Camera mounted on UAV can be regarded as a visual sensor for collecting aerial videos. Compared with traditional sensors, the UAV can be flexibly deployed to the areas that need to be monitored and can provide a larger perspective. In this paper, a novel framework for vehicle counting based on aerial videos is proposed. In our framework, the moving-object detector can handle the following two situations: static background and moving background. For static background, a pixel-level video foreground detector is given to detect vehicles, which can update background model continuously. For moving background, image-registration is employed to estimate the camera motion, which allows the vehicles to be detected in a reference coordinate system. In addition, to overcome the change of scale and shape of vehicle in images, we employ an online-learning tracker which can update the samples used for training. Finally, we design a multi-object management module which can efficiently analyze and validate the status of the tracked vehicles with multi-threading technique. Our method was tested on aerial videos of real highway scenes that contain fixed-background and moving-background. The experimental results show that the proposed method can achieve more than 90% and 85% accuracy of vehicle counting in fixed-background videos and moving-background videos respectively.

## 1. Introduction

With the rapid development of intelligent video analysis, traffic monitoring has become a key technique for collecting information about traffic conditions. Using the traditional sensors such as magnetometer detectors, loop detectors, ultrasonic sensors, and surveillance video cameras may cause damage to the road surface [1,2,3,4]. Meanwhile, because many of these sensors need to be installed in urban areas, the cost of this work is high. Among them, surveillance video cameras are commonly used sensors in the traffic monitoring field [5,6,7], which can provide video stream for vehicle detection and counting. However, there are many challenges for using surveillance video cameras, such as occlusion, shadows, and limited view. To address these problems, Lin [8] resolved the occlusion problem with occlusion detection and queue detection. Wang [9] detected shadows based on shadow characteristics such as lower lightness and the lack of textures. Douret [10] used a multi-camera method to cover large areas for avoiding occlusion. In [11], two omnidirectional cameras are mounted on vehicle, performing binocular stereo matching on the rectified images to obtain a dynamic panoramic surround map of the region around the vehicle. Further, many researchers apply vehicle detection and tracking to vehicle counting. Srijongkon [12] proposed a vehicle counting system based on ARM/FPGA processor, which uses adaptive background subtraction and shadow elimination to detect moving vehicle and then counts the vehicles in video screen. Prommool [13] introduced a vehicle counting framework using motion estimation (block matching and optical flow combination). In [13], the area box is set at the intersection to determine whether the vehicle passes through. Swamy [14] presented a vehicle detection and counting system based on color space model, which uses color distortion and brightness distortion of image to detect vehicle and then counts vehicle using a pre-defined line. Seenouvong [15] used background subtraction method to detect foreground vehicles in surveillance video sequence and calculates centroid of objects in virtual detection zone for counting. Although researchers improve the traditional traffic monitoring methods and apply vehicle detection and tracking on vehicle counting, the traditional surveillance camera still cannot be applied for monitoring large areas, which is very restrictive for vehicle counting. Besides, the research in [12,13,14,15] does not use a multi-object management system to confirm the uniqueness of vehicles, which is unreliable for long sequence vehicle counts and effects the efficiency of vehicle counting. In contrast to traditional traffic monitoring sensors, the UAV can be flexibly deployed to the regions that need to be monitored. Moreover, the UAV is a cost effective platform that can monitor a large continuous stretch of roadway and can focus on a specific road segment. In addition, to focus on large area monitoring, the UAV provides a wider top-view perspective. By achieving a large top-view perspective, the UAV can provide efficient data acquisition for intelligent video analysis.

In recent years, several methods for traffic monitoring from aerial video are presented. Ruimin Ke [16] developed an approach for vehicle speed detection by extracting interest points from a pair of frames and performs interest-point tracking from aerial videos by applying Kanade–Lucas optical flow algorithm. Shastry [17] proposed a video-registration technique for detecting vehicles using KLT (Kanade–Lucas–Tomasi) features tracker to automatically estimate traffic flow parameter from airborne videos. Cao [18] proposed a framework for UAV-based vehicle tracking using KLT features and a particle filter. The research in [16,17,18] uses KLT features tracker which detects moving-object by extracting optical flow of interest points and can be used in case of moving background. The interest points are used to efficiently extract the feature of interest region, which can reduce the amount of computation. However, because of the complexity of scene in aerial videos, some background points may be extracted as interest points, which brings noises to the subsequent tracker. Pouzet [19] proposed a real-time method for image-registration dedicating to small moving-object detection from a UAV. The techniques in [17,19] are both equipped with image-registration, which segments the moving vehicles by transforming the previous frame to the current frame from aerial videos. Image-registration allows for the comparison of the images in a reference frame, so the scene can be analyzed in a reference coordinate system. Freis [20] described an algorithm for the background-subtraction based vehicle-tracking for vehicle speed estimation using aerial images taken from a UAV. Chen [21] proposed a vehicle detection method from UAVs which integrated of Scalar Invariant Feature Transform(SIFT) and Implicit Shape Model(ISM). Guvenc [22] proposed a review paper for object detection and tracking from UAVs. Shi [23] proposed a moving vehicle detection method in wide area motion imagery, which constructs a cascade of support vector machine classifiers for classifying object and can extract road context. Further, LaLonde [24] proposed a cluster network for small object detection in wide area motion imagery, which combines both appearance and motion information. However, the research in [23,24] focuses on small object detection in wide area motion imagery which is captured at very high altitude and is hard to capture with an universal UAV. For vehicle counting from UAV, Wang [25] proposed a vehicle counting method with UAV by using block sparse RPCA algorithm and low rank representation. However, the UAV only works on hovering mode and captures static background images. In addition, without a multi-object tracking and management module, the method cannot distinguish the direction and uniqueness of the vehicle, which can easily lead to counting error.

In this paper, a multi-vehicle detection and tracking framework based on UAV is proposed, which can be used for vehicle counting and can handle both fixed-background and moving-background. First, the UAV collects the image sequence and transmits it to the detector which is divided into two parts: static background and moving background. To confirm the unique identity of the vehicles in long sequence video, all detected vehicles are tracked by the tracker. To manage the tracked vehicles efficiently and avoid tracking chaos, we design a multi-object management module which manages the tracked vehicles under a unified module and provides status information of each tracked vehicle for subsequent intelligent analysis. In addition, to improve the computational efficiency, we incorporate parallel processing technology into the multi-objective management module. In summary, our method mainly includes four components: moving-vehicle detection, multi-vehicle tracking, multi-vehicle management module, and vehicle counting.

The rest of this paper is organized as follows. Section 2 describes the architecture of the vehicle counting system from aerial videos. Section 3.1 focuses on vehicle detection. Section 3.2 and Section 4 mainly discuss the tracking algorithm framework and multi-object management module. Section 5 mainly introduces the vehicle counting module. In Section 6, we present the experimental results. Finally, we give a brief conclusion in Section 7.

## 2. Architecture of the System

**Figure 1:**
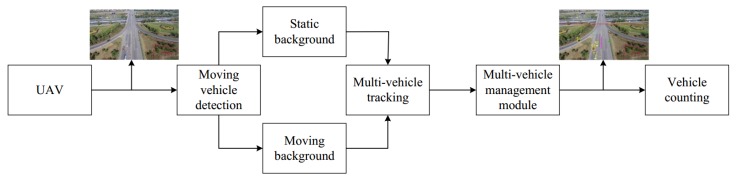
Framework of the proposed method. It consists of vehicle detection, multi-vehicle tracking, multi-vehicle management, and vehicle counting. The UAV is equipped with a visual sensor. Vehicles are detected by the detector which can handle two situations: static background and moving background. Then, the detected vehicles are tracked by tracking module. By analyzing the results of the tracker, we can count the number of vehicles.

Our framework is based on the platform of UAV. In Figure 1, the video stream is captured by a camera mounted on the UAV. The detector can deal with two situations(static background and moving background). We distinguish whether the background is moving or not according to the motion mode of the UAV. For static background, a samples-based algorithm for background subtraction is employed in our framework, which can detect moving vehicle by modeling background and can update the background model continuously. By updating the background model, the parameters of model are more suitable to describe the real-time scene. For moving background, the camera platforms move with UAV. In this case, image-registration is used in our framework to transform the camera coordinates of adjacent frames to a reference coordinate system. Thus, the movement of camera can be compensated in the adjacent frames, so that we can detect vehicles from the reference frame. Images captured by UAV are characterized by complex background and variable vehicle shape, which leads to discontinuity of detector, and thus affects the accuracy of vehicle counting. Thus, to address this problem, an online-learning tracker is used in our framework, which can update the samples used for training. Further, considering that traditional tracker can only track one object, we design an efficient multi-object management module by using multi-threading technology, which can assign multi-object tracking task to parallel blocks and can analyze and validate the status of the tracked vehicles. Finally, the status of the tracked vehicles is used to count the number of vehicles.

## 3. Vehicle Detection and Tracking

### 3.1. Vehicle Detection

#### 3.1.1. Static Background

Vehicle detection is an essential process for vehicle counting. In this section, we mainly discuss how the UAV works in hovering mode. In the case of fixed background, we can extract moving vehicles by using background modeling. ViBe [26] for vehicle detection is employed in our framework with the following advantages. One of the advantages of the ViBe foreground detection algorithm is that the background model can be updated. By updating the background model, the noise points caused by slight variations of brightness can be effectively suppressed in images. Another advantage is that ViBe first selects a certain area in image for background modeling, rather than modeling the entire image, which greatly reduces the computational load.

**Figure 2:**
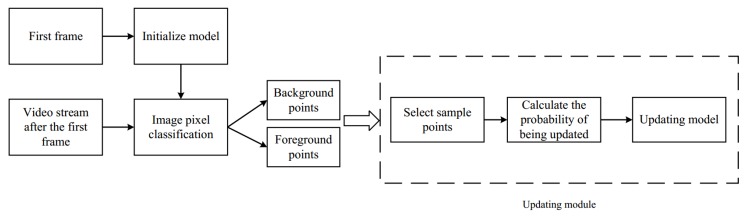
The overview of ViBe algorithm. Given a UAV video stream, the first step of ViBe is to initialize the background. After initialization of the background model at the first frame, the algorithm begins extracting foreground at the second frame. For updating model, sample points are selected randomly, and then the probability of updating is calculated.

An overview of ViBe algorithm is given in Figure 2. The first step of ViBe is to initialize the background. Each background pixel is modeled by a collection of *N* background sample values $\left[v_{1},v_{2},\ldots ,v_{N}\right]$. We randomly select the pixel values of its neighbours as its modeling sample values. To classify the pixel v(x), a difference *D* between pixel values in the field centered at the point v(x) is defined. The value of *D* for gray image is defined in Equation (1):

(1) $$D=\left|v\left(x\right)-v_{i}\right|,$$

and *D* for RGB image is

(2) $$D=\left|v_{r}\left(x\right)-v_{ri}\right|+\left|v_{g}\left(x\right)-v_{gi}\right|+\left|v_{b}\left(x\right)-v_{bi}\right|.$$

The $v_{i}$ in Equation (1) is a background sample value. In Equation (2), the center pixels $v_{r}\left(x\right)$, $v_{g}\left(x\right)$, and $v_{b}\left(x\right)$ correspond to three channels. The $v_{ri}$, $v_{gi}$, and $v_{bi}$ are background sample values corresponding to three channels. We use the gray-scale image as an example to analyze the principles of the algorithm. Here, three parameters about pixels classification are defined. $D_{t}$ is the pixel difference min threshold. *S* and $S_{t}$ are the number of points above the pixel difference min threshold $D_{t}$ and the min value of *S*. If $S>S_{t}$, the point $v_{x}$ is classified into background.

To improve the detection performance on moving objects under background changes, an updating model method is employed. In the method, the probability of updating each background point is 11φφ. The probability of updating neighbour’s points is 11φφ. Updating the neighbour’s sample pixel values takes advantage of the spatial propagation characteristics of the pixel values. Then, the background model gradually diffuses outwards. When a pixel is judged to be a background point, the probability of updating the background model is 11φφ. In general, updating process is composed of three steps: randomly selecting the sample update, randomly deciding whether to update the background model, and randomly deciding whether to update the field pixels.

#### 3.1.2. Moving Background

**Figure 3:**
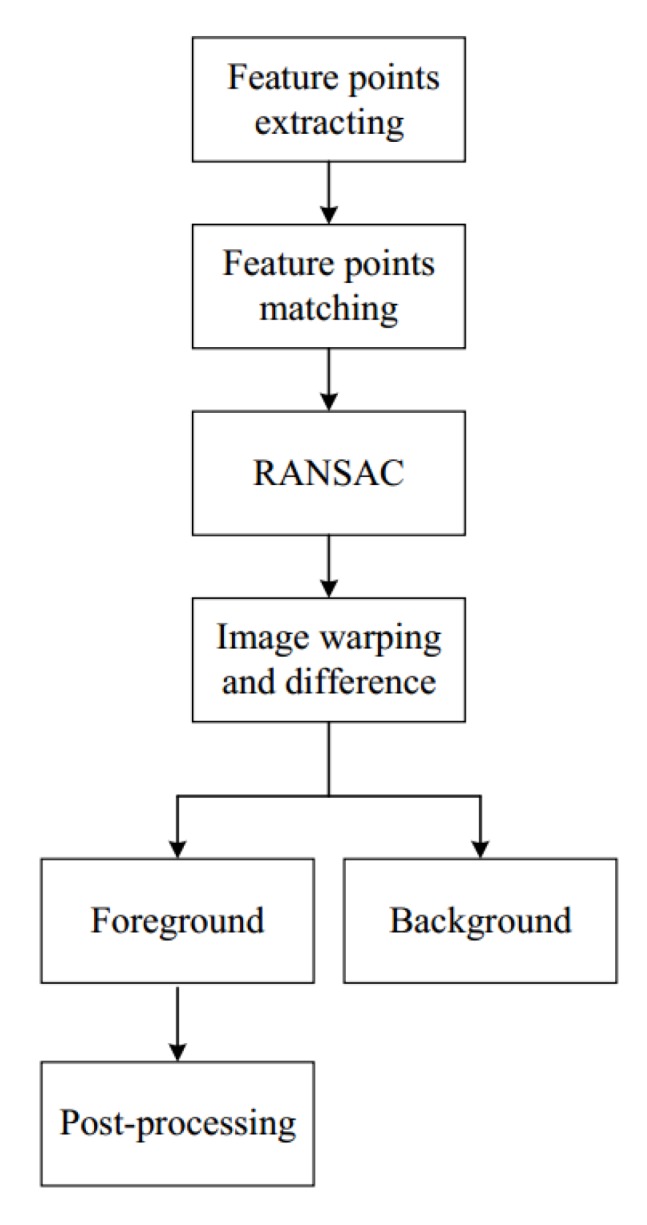
Moving background detector. SURF feature points are extracted firstly, which are used to match two frames. RANSAC algorithm is employed to estimate the transformation between the two frames. After that, we transform the camera coordinates of adjacent frames to a reference coordinate system. Then, image difference method is used to extract foreground. The final results are processed by morphological method.

In this section, we mainly discuss how the UAV works in moving mode. The overview of this section is shown in Figure 3. SURF feature [27] points are extracted to describe the features of frames. We use fast approximate nearest neighbour search approach to match the feature points. We aim at finding a transformation *W* which can warp the image $I_{t}$ to image $I_{t+1}$. We assume the eight-parameter transformation *W* is the following:

(3) $$W=\left[\begin{array}{ccc} m_{1} & m_{2} & m_{3}\\ m_{4} & m_{5} & m_{6}\\ m_{7} & m_{8} & 1 \end{array}\right],$$

where the $m_{1}$, $m_{2}$, $m_{3}$, $m_{4}$, $m_{5}$, $m_{6}$, $m_{7}$, and $m_{8}$ are parameters of warping. We can define the final transformation formula as follows:

(4) $$\left[\begin{array}{c} x^{\prime }\\ \begin{array}{c} y^{\prime }\\ 1 \end{array} \end{array}\right]=W\left[\begin{array}{c} x\\ y\\ 1 \end{array}\right]\;$$

where (x,y) and $\left(x^{\prime },y^{\prime }\right)$ are pixel points on $I_{t}$ and the warped image $I^{\prime }$, respectively. To estimate *W*, we assume that the transformation between frames can be modeled by a homography and use the Random Sample Consensus (RANSAC) algorithm [28].

After estimating the warped image $I^{{}^{\prime }}$, we use the image difference method to extract the moving vehicle,

(5) $$\delta =I^{\prime }\left(x^{\prime },y^{\prime }\right)-I\left(x,y\right),$$

where the δ denotes the pixel difference value of each point of image *I*. We set μ as the threshold of δ. If δ>μ, we determine that the point (x,y) is foreground point. To partially suppress the ghost problem, we conduct morphological post-processing for foreground objects. During this process, foreground objects are dilated and then eroded to suppress noise around the object.

### 3.2. Vehicle Tracking

Compared with other tracking-by-detection methods, such as TLD [29] and STRUCK [30], the speed of KCF [31] has been greatly improved. Because of the complexity of ground conditions in UAV videos, the multi-scale and shape changes of vehicles will affect the effect of tracker. To address this issue, we employ the online-learning tracker, which considers the process of tracking as a ridge regression problem and trains a detector in tracking process. The detector is used to detect the location of the object in the next frame. During training, the inputs are samples and labels, such as $\left(x_{1},y_{1}\right),\left(x_{2},y_{2}\right),\ldots ,\left(x_{n},y_{n}\right)$. To determine the label value $y_{i}$, which is a number in [0,1], we calculate the distance between the object center and the sample center. If the sample is close to the object, $y_{i}$ tends to 1, and if not tends to 0. The goal of training is to find a function $f\left(z\right)=w^{T}z,z=\left[z_{1},\ldots ,z_{n}\right]$ that minimizes the squared error over samples,

(6) $$\min_{w}\sum_{i}{\left(f\left(x_{i}\right)-y_{i}\right)}^{2}+\lambda {∥w∥}^{2},$$

where λ is a regularization parameter that controls over-fitting.

**Figure 4:**
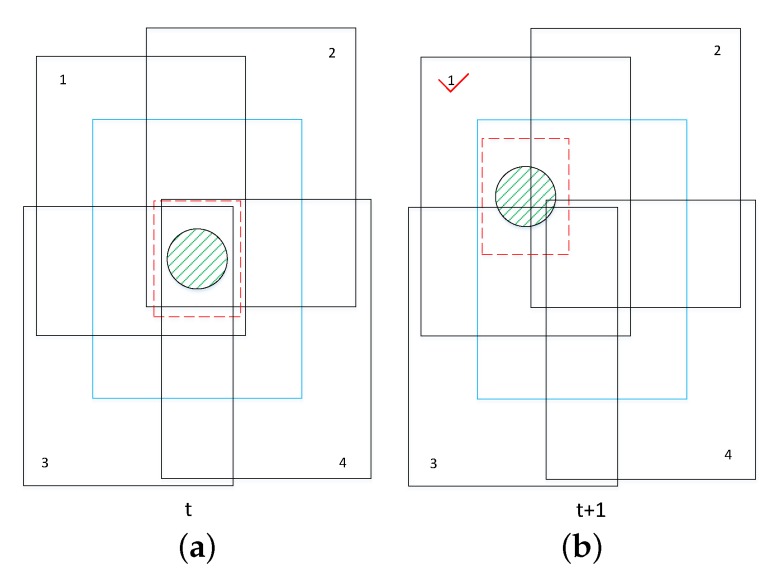
Tracking process diagram. It shows that, during tracking process, we train a regression by finding samples near the object in frame *t* and use the regression to estimate the displacement of the tracked object in frame t+1: (**a**) the object’s state in frame *t*; and (**b**) the object’s state in frame t+1. The tick symbol means that No. 1 box receives responses the most.

The KCF tracking process can be mainly divided into the following steps. First, for frame *t*, a classifier is trained using the tracker samples selected near the prior position $P_{t}$, which calculate the response of a small window sample. Then, in frame t+1, samples are obtained near the previous frame’s position $P_{t}$, and the response of each sample is judged by the classifier trained in frame *t*. The strongest response of the sample is the predicted position $P_{t+1}$. As shown in Figure 4, in frame *t*, the red dashed box is the initial tracking box which is expanded by a factor of 2.5 as a prediction box (blue). The black boxes around the object are sample boxes obtained after the blue box has been cyclically shifted. We use these sample boxes to train a classifier. In frame t+1, we first sample in the predicted area, that is, the blue solid-line box area. Then, we use the classifier to calculate the responses of these boxes. Obviously, the No. 1 box receives responses the most. Thus, we can predict the displacement of the object.

There are two options for extracting the features of object: one is the gray feature and the other is the HOG feature [32]. Here, we use the HOG feature.

## 4. Multi-Vehicle Management Module

**Figure 5:**
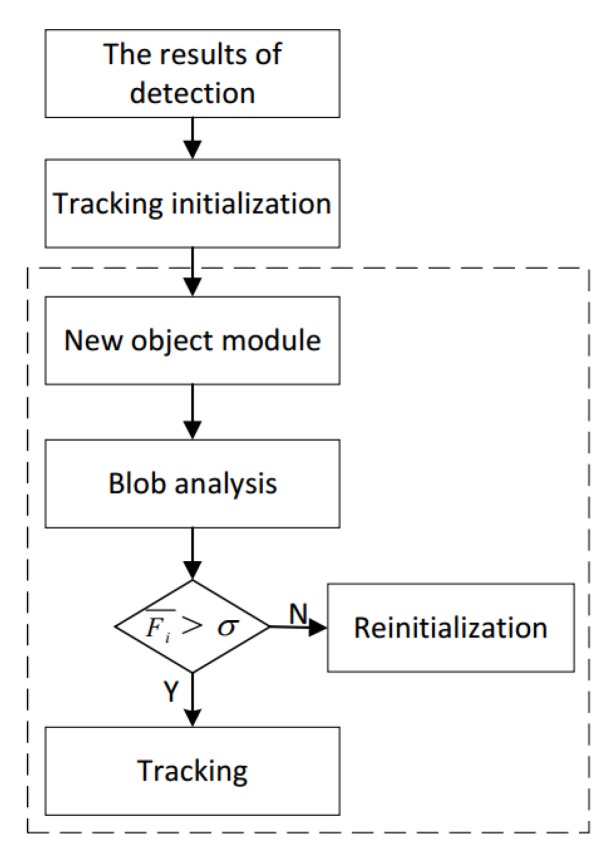
Multi-vehicle management module. Inside the dashed box is tracking module which connects detector and tracker. It consists of new object module, blob analysis and multi-object tracking.

**Figure 6:**
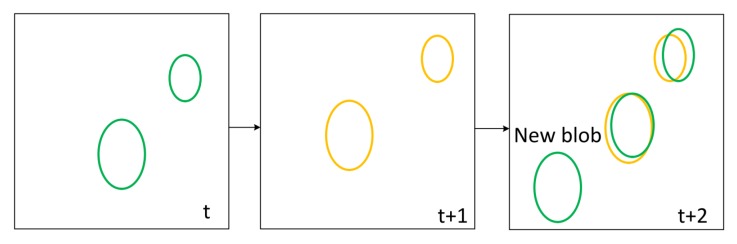
New-object identification. Two blobs (green) are detected in frame *t*. In frame t+1, two blobs (yellow) are tracked. Then, a blob (green) is classified as new blob in frame t+2, which will be added to tracker.

**Figure 7:**
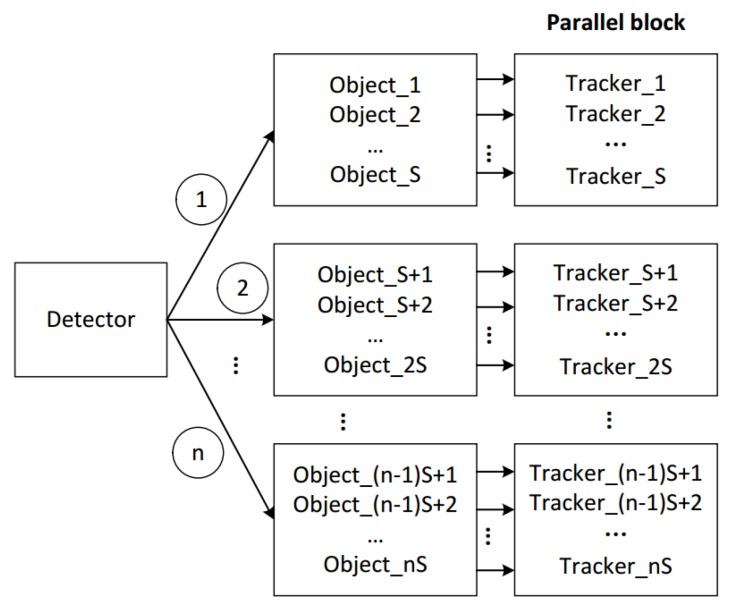
The multi-object tracker. The results of detector are processed sequentially in parallel blocks containing different numbered trackers. *S* denotes CPU kernel number. The numbers 1 to *n* are the order of processing.

Considering that the original KCF tracker can only track one object, we design a multi-vehicle management module by using multi-threading technology, which can assign multi-object tracking task to parallel blocks and can efficiently analyze and validate the status of the tracked vehicles. We assume that the objects detected by the detector are $O_{1},O_{2},\ldots ,O_{n}$. As shown in Figure 5, first, the detection results are all given to the tracker for initialization. We present the initialized objects as $O_{1}^{i},O_{2}^{i},\ldots ,O_{n}^{i}$. After that, the detected vehicles $O_{1},O_{2},\ldots ,O_{n}$ in each frame are given to the new object module to determine. We describe the new object module with n=2. As shown in Figure 6, in frame *t*, we detect two new blobs $O_{1d}^{{}^{\prime }}$ and $O_{2d}^{{}^{\prime }}$ represented by green ellipses. In frame t+1, we use two yellow ellipses to represent the two blobs $O_{1t}^{{}^{\prime }}$ and $O_{2t}^{{}^{\prime }}$ that have been tracked. In frame t+2, by analyzing the overlap between the detection box and the tracking box, a new blob $O_{new}^{{}^{\prime }}$ can be determined by new object module. In our experiments, we use γ to indicate the overlap ratio between tracking box and detection box. If γ<0.1, the new blob will be added to the tracker. We denote the final tracked objects as $O_{1}^{t},O_{2}^{t},\ldots ,O_{m}^{t}$. In fact, it can be time consuming for algorithm to handle multiple objects. To address this problem, we design a multi-objective processing mode of recurrent multi-thread. Each object can be automatically assigned to a separate thread to process. At the same time, the system allocates separate memory space to each object. If the target disappears, the algorithm automatically retrieves the thread and the corresponding memory space, which are provided for subsequent new object to use. In this way, threads can be allocated and reclaimed in parallel, which can deal with multiple objects efficiently. In Figure 7, the results of detector $O_{1},O_{2},\ldots ,O_{n}$ are processed by different trackers that are handled by different threads. *S* threads are divided into one block and the whole thread network is composed of multiple thread blocks. By applying the multi-threading technology, the computational load is greatly reduced.

In the multi-vehicle management module, all errors of trackers are analyzed according to the response of regression to avoid these errors in the future. We define the response of regression as $F_{ti}$, where *i* is the blob number and *t* is the frame number. During tracking, the average regression response of the blob *i* can be expressed as following,

(7) $${\bar{F}}_{i}=\sum_{n=1}^{N}F_{ni},$$

where *N* is the current total number of frames. We define the confidence threshold of the blob as σ. If $\bar{F_{i}}>\sigma$, blob *i* will be tracked continuously. If $\bar{F_{i}}\leq \sigma$, blob *i* will be reinitialized by detector. The final tracked results are used to count vehicles. We mainly discuss the vehicle counting module in the next section.

## 5. Vehicle Counting Module

**Figure 8:**
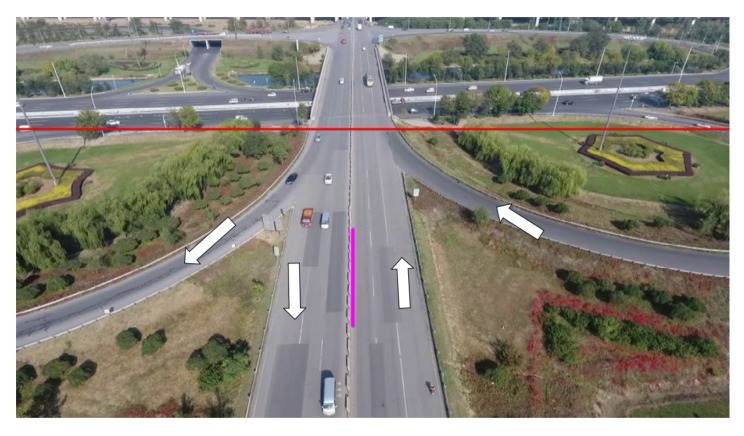
Vehicle-counting area and direction. Below the red line is the detection area, in which the right side of the pink line is the forward direction, and the left side is the backward direction.

The commonly used vehicle counting method is based on the regional mark and the virtual test line. The former method is to count the number of connected areas, while the latter sets up a virtual test line on the road. We define an area that is used to count vehicles. We count the vehicles in the area below the red line. On the highway, we divide the vehicles into two directions as shown in Figure 8. Because our method is equipped with multi-vehicle tracking and management modules, there is no need to set up multiple lines in the area to determine whether the vehicles are passing. In the multi-vehicle management module, the information of ID and direction of the vehicles are stored, which can be used to directly count the vehicles in the region. For example, we assume the number of vehicles tracked at frame *t* is *m*. If a vehicle is tracked at frame t+1 with a different ID, then we determine the counter plus 1.

In summary, the proposed vehicle counting method is based on the multi-object management module assembling the detectors and trackers to work together in an unified framework. Each tracker tracks an independent object with no interference between the objects, which ensures that the status information of each object is not confused. When the result of the tracker is unreliable, the detector reinitializes the corresponding tracker. In terms of multiple tracker processing, we employ multi-threading technology, which can greatly reduce the computational load.

## 6. Evaluation

In this section, we provide the results of a real-world evaluation of our method. The method was implemented with C++ and OpenCV. We tested our algorithm on a system with an Intel Core i5-4590-3.30 GHz CPU, 8G memory and Windows 10 64-bit operating system.

### 6.1. Dataset

In our experiments, we used a UAV to record the real freeway scene videos at 960 × 540 resolution. The data were divided into two groups, one was the height of 50 m and the other was the height of 100 m. The flight modes of the UAV were set to two types in our experiments, static hovering and linear horizontal flight. Table 1 shows the details of the test videos.

### 6.2. Estimation Results and Performance

**Figure 9:**
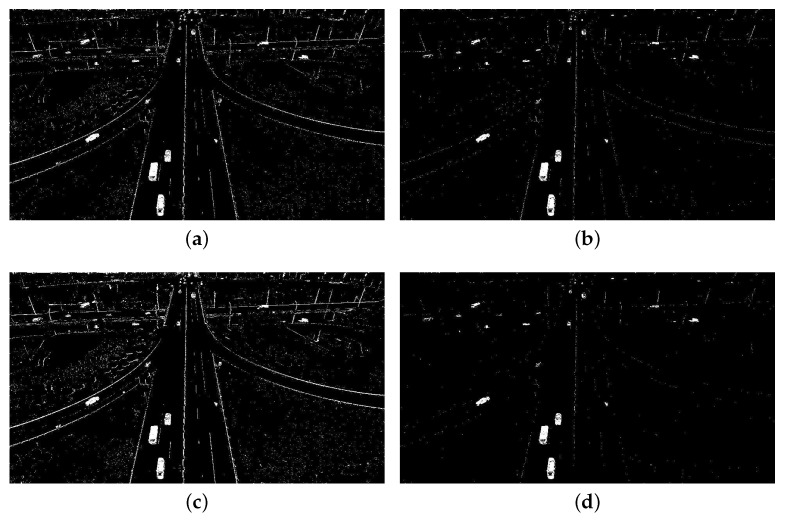
Examples showing the influence of parameter settings of detector (static background). Four combinations of *N* and $S_{t}$ were tested, showing the influence of parameters on vehicle detection: (**a**) $N=10,\;S_{t}=2$; (**b**) $N=30,\;S_{t}=2$;. (**c**) $N=50,\;S_{t}=6$; and (**d**) $N=50,\;S_{t}=2$.

**Figure 10:**
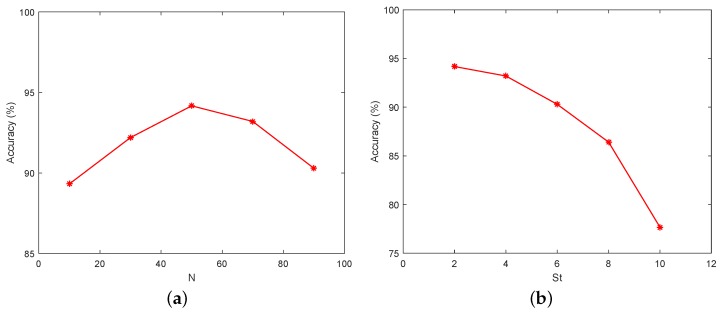
Accuracy of vehicle counting with different parameters (static background). We tested our method on a video of static background scene with different parameters: (**a**) the effect of *N* on accuracy when the value of $S_{t}$ is fixed to 2; and (**b**) the effect of $S_{t}$ on accuracy when the value of *N* is fixed to 50.

**Figure 11:**
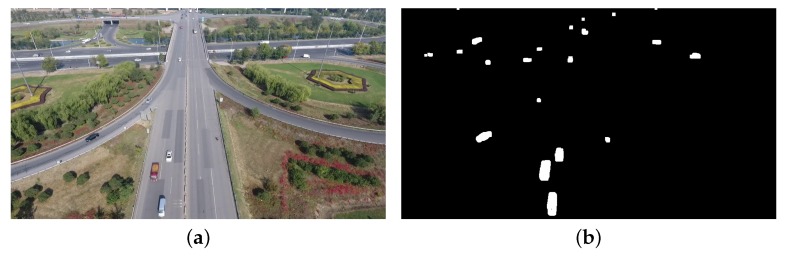
An example of post-processing(static background). Median filter is used to post-process the image. To suppress the noise contained in the segmented image, we chose the area of the object to be filtered: (**a**) the original image; and (**b**) the final result of detector.

For static background, the moving vehicles were detected from each frame using the Vibe algorithm. The settings of parameters in our experiments are displayed in Table 2. The first parameter we set is the number of samples *N*, which is related to the resolution of the image and the average size of the vehicles. Thus, if *N* is set too small, many background points will be mistakenly detected as a foreground points. Some noises that are not vehicles will be detected as vehicles, because *N* affects the background model and the sensitivity of the model. On the other hand, if *N* is too large, the processing speed will be reduced. The parameters min match value $S_{t}$ and the pixel difference min threshold $D_{t}$ are also related to the model and affect the sensitivity of the model. The last parameter update factor φ determines the updating speed of the background, which is inversely proportional to the updating speed of the background. An example showing the influence of these parameters is presented in Figure 9. Comparing Figure 9a,b, we can note that the smaller parameter *N* resulted in many noise points in the background. Moreover, the larger value of $S_{t}$ also led to many noise points in background, as shown in Figure 9c,d. Obviously, the parameters of detector affect the results of detection. Further, we set different parameters to test the accuracy of the vehicle counting on TEST_VIDEO_1. Figure 10 shows the effect of different parameter settings on accuracy. In Figure 10, we can find that when *N* is 50 and $S_{t}$ is 2, the highest precision is achieved. Hence, setting proper parameter values is important to accuracy. Figure 11 shows that after the morphological processing, the results of segment are more complete.

**Figure 12:**
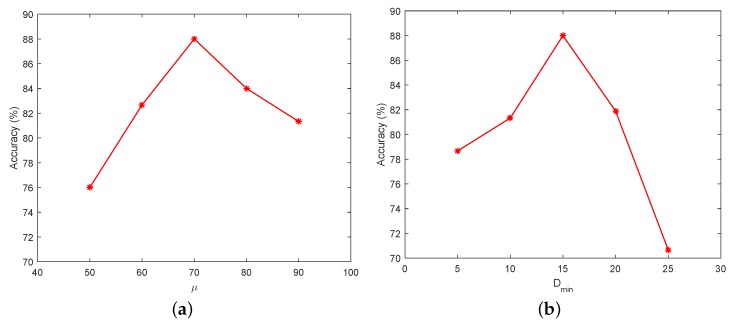
Accuracy of vehicle counting with different parameters (Moving background). We tested our method on a video of moving background scene with different parameters: (**a**) the effect of μ on accuracy when the value of $D_{min}$ is fixed to 15; and (**b**) the effect of $D_{min}$ on accuracy when the value of μ is fixed to 70.

**Figure 13:**
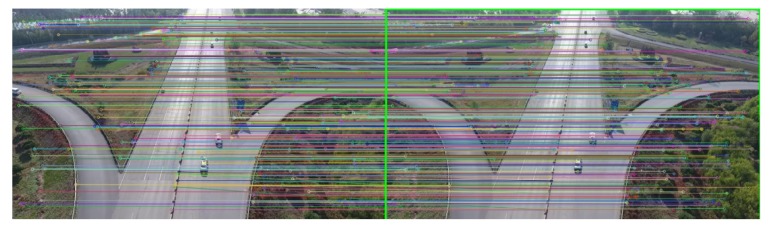
The matching map of two consecutive images. We used different colors to represent the corresponding matching points, and connected these points with different lines.

**Figure 14:**
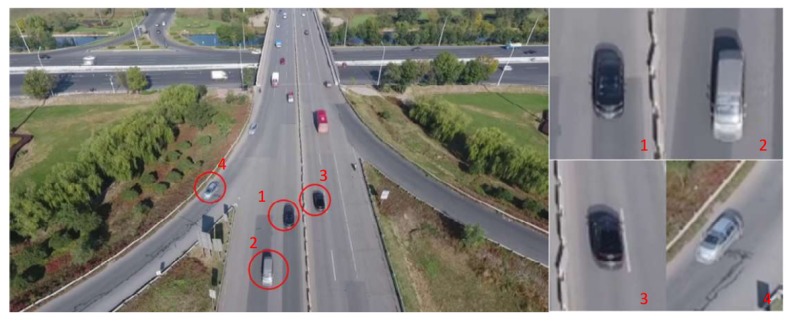
The overlaid image of the warped image and the reference image. The weights of the warped image and the reference image are each 0.5. The left image is the overlaid image. We circle four vehicles as examples to show the results of warping. The right four images are large vision of the circle vehicles which are represented in a reference coordinate system.

**Figure 15:**
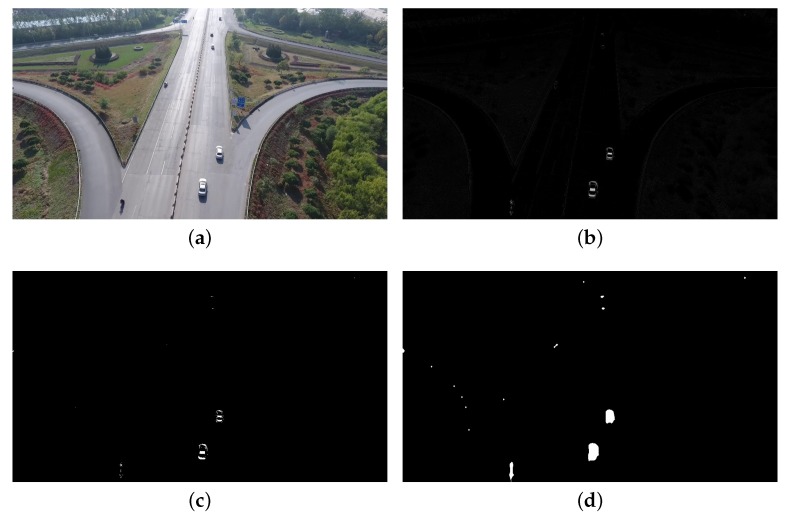
The results of vehicle detection(moving background): (**a**) a selected frame of video stream; (**b**) initial difference image; (**c**) the result of segment; and (**d**) the result of post-processing.

For moving background, *H* denotes the threshold of the response of the determinant of Hessian matrix. $D_{min}$ is the distance threshold of matching point. To analyze the effect of parameter setting on accuracy, we tested the vehicle counting accuracy on TEST_VIDEO_5 with different parameter settings. Figure 12 shows the effect of μ and $D_{min}$ on accuracy. Because μ represents the threshold of image segmentation, the results of segmentation are greatly affected, which in turn will affect the accuracy of the vehicle counting. In Figure 12b, the value of $D_{min}$ greatly affects the precision of counting, especially when the value of $D_{min}$ is too high. This is because $D_{min}$ controls the search range of matching points, which directly affects the accuracy of image registration. An example of the result of matching is shown in Figure 13. In Figure 14, the warped image is overlaid on the reference image. To detect foreground, we calculated the difference between the warped image and the reference image. The results of vehicle detection are shown in Figure 15.

**Figure 16:**
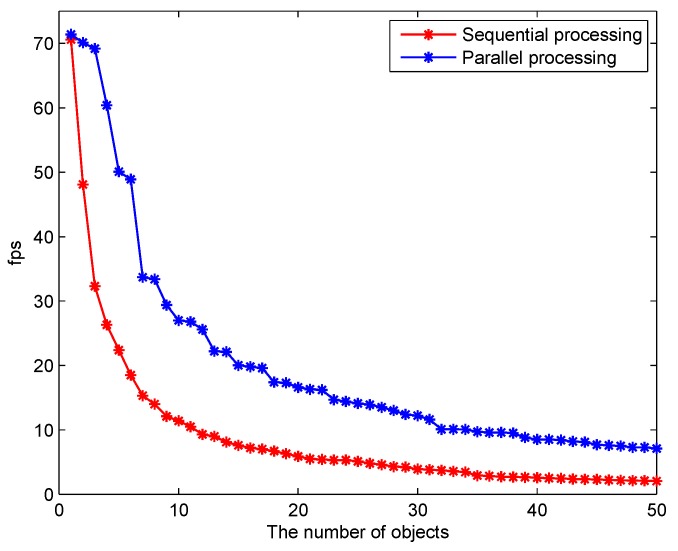
Processing speed comparison (tracker). This figure shows the relationship between the processing speed and the total number of objects (sequential and parallel processing).

During tracking, the confidence threshold of blob σ, area-expansion factor padding and the cell size of the HOG feature cell need to be set, which are shown in Table 2. To test the performance of tracker in tracking multiple objects, we recorded the processing speed of tracking 1–50 objects simultaneously. In Figure 16, the parallel tracker shows obvious superiority relative to traditional sequential processing in terms of processing speed.

**Figure 17:**
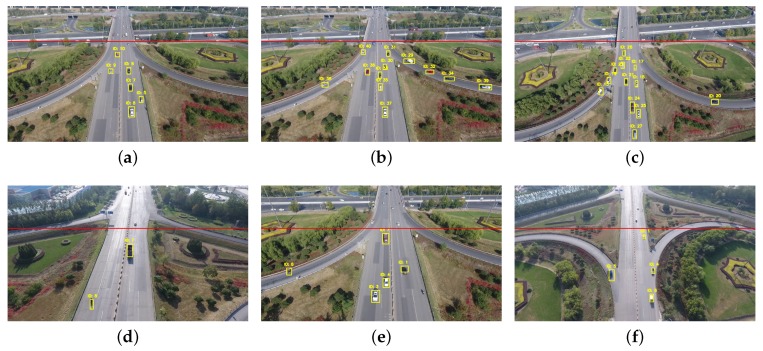
The vehicle tracking results on test aerial video: (**a**–**c**) captured with camera fixed; (**d**–**f**) captured with camera moving; (**a**,**b**,**d**,**e**) captured at a height of 50 m; and (**c**,**f**) captured at a height of 100 m.

The summary of the estimation results and performance are presented in Table 3. We used eight videos at the height of 50 m and eight videos at the height of 100 m to test our method. The used test videos are described in Section 6.1. As shown in Figure 17, we selected six frames from the test videos to show the final results, which were collected at heights of 50 m and 100 m. Test videos were manually examined frame by frame to obtain the ground-truth values of vehicle counting. ε denotes the accuracy rate,

(8) $$\varepsilon =\frac{N_{estimated}}{N_{truth}}\times 100\%,$$

where $N_{estimated}$ and $N_{truth}$ denote the estimated value and ground truth. In Table 3, the average error rate for the height of 50 m are less than those for the height of 100 m, because some small objects are regarded as background by detector. The accuracy of the static background is higher than the accuracy of the moving background, which indicates that the error of the estimation of camera motion can affect the results of vehicle detecting and the final results. By considering the results of the analyses above, we can conclude that our method works well on both moving-background aerial videos and fixed-background aerial videos and can achieve more than 90% and 85% accuracy of vehicle counting, respectively.

## 7. Conclusions

In this paper, an efficient vehicle counting framework based on vehicle detection and tracking from aerial videos is proposed. Our method can handle two situations: static background and moving background. For static background, we employ a foreground detector which can overcome the slight variations of real scene by updating model. For moving background, image-registration is used to estimate the camera motion, which allows detecting vehicle in a reference frame. In addition, to address the change of shape and scale of vehicle in images, an online-learning tracking method is employed in our framework, which can update the samples used for training. In particular, we design a multi-object management module which can connect the detector and the tracker efficiently by using multi-threading technology and can intelligently analyze the status of the tracked vehicle. The experimental results of 16 aerial videos show that the proposed method yields more than 90% and 85% accuracy on fixed-background videos and moving-background videos, respectively.

## Figures and Tables

**Table 1:** The information of test data.

Aerial Videos	Height	Static Background	Moving Background	Total Number of Frames
TEST_VIDEO_1	50	*√*		5638
TEST_VIDEO_2	50	*√*		5770
TEST_VIDEO_3	50	*√*		5729
TEST_VIDEO_4	50	*√*		5820
TEST_VIDEO_5	50		*√*	5432
TEST_VIDEO_6	50		*√*	5533
TEST_VIDEO_7	50		*√*	5573
TEST_VIDEO_8	50		*√*	5599
TEST_VIDEO_9	100	*√*		5920
TEST_VIDEO_10	100	*√*		5733
TEST_VIDEO_11	100	*√*		5527
TEST_VIDEO_12	100	*√*		5573
TEST_VIDEO_13	100		*√*	5620
TEST_VIDEO_14	100		*√*	5734
TEST_VIDEO_15	100		*√*	5702
TEST_VIDEO_16	100		*√*	5523

**Table 2:** Parameter settings.

Parameters	Height		Background	
	50	100	Fixed	Moving
*N*	50	45	*√*	-
$D_{t}$	15	13	*√*	-
$S_{t}$	2	2	*√*	-
φ	5	5	*√*	-
μ	70	60	-	*√*
*H*	100	100	-	*√*
$D_{min}$	15	10	-	*√*
padding	2.5	2	*√*	*√*
cell	4×4	4×4	*√*	*√*
σ	0.2	0.3	*√*	*√*

**Table 3:** Accuracy analysis on test video.

Height	Direction	Total Number of Vehicles	Number of the Counted Vehicles	Accuracy	Background
50	Forward	202	193	95.54%	Fixed
50	Backward	217	207	95.39%	Fixed
50	Forward	164	144	87.80%	Moving
50	Backward	139	122	87.77%	Moving
50	Forward and background	722	666	92.24%	Fixed and moving
100	Forward	174	160	91.95%	Fixed
100	Backward	238	219	92.02%	Fixed
100	Forward	173	148	85.55%	Moving
100	Backward	147	126	85.71%	Moving
100	Forward and backward	732	653	89.21%	Fixed and moving
50 and 100	Forward and backward	831	779	93.74%	Fixed
50 and 100	Forward and backward	623	540	86.68%	Moving
50 and 100	Forward and backward	1454	1319	90.72%	Fixed and Moving

## References

[1] Scarzello J., Lenko D., Brown R., Krall A. (1978). SPVD: A magnetic vehicle detection system using a low power magnetometer. IEEE Trans. Magnet..

[2] Ramezani A., Moshiri B. The traffic condition likelihood extraction using incomplete observation in distributed traffic loop detectors. Proceedings of the 2011 14th International IEEE Conference on Intelligent Transportation Systems (ITSC).

[3] Agarwal V., Murali N.V., Chandramouli C. (2009). A Cost-Effective Ultrasonic Sensor-Based Driver-Assistance System for Congested Traffic Conditions. IEEE Trans. Intell. Transp. Syst..

[4] Lu X., Ye C., Yu J., Zhang Y. A Real-Time Distributed Intelligent Traffic Video-Surveillance System on Embedded Smart Cameras. Proceedings of the 2013 Fourth International Conference on Networking and Distributed Computing.

[5] Ebrahimi S.G., Seifnaraghi N., Ince E.A. Traffic analysis of avenues and intersections based on video surveillance from fixed video cameras. Proceedings of the 2009 IEEE 17th Signal Processing and Communications Applications Conference.

[6] Thadagoppula P.K., Upadhyaya V. Speed detection using image processing. Proceedings of the 2016 International Conference on Computer, Control, Informatics and its Applications (IC3INA).

[7] Engel J.I., Martín J., Barco R. (2017). A Low-Complexity Vision-Based System for Real-Time Traffic Monitoring. IEEE Trans. Intell. Transp. Syst..

[8] Lin S.P., Chen Y.H., Wu B.F. A Real-Time Multiple-Vehicle Detection and Tracking System with Prior Occlusion Detection and Resolution, and Prior Queue Detection and Resolution. Proceedings of the 18th International Conference on Pattern Recognition (ICPR’06).

[9] Wang J.M., Chung Y.C., Chang C.L., Chen S.W. Shadow detection and removal for traffic images. Proceedings of the IEEE International Conference on Networking, Sensing and Control.

[10] Douret J., Benosman R. A volumetric multi-cameras method dedicated to road traffic monitoring. Proceedings of the IEEE Intelligent Vehicles Symposium.

[11] Gandhi T., Trivedi M.M. (2006). Vehicle Surround Capture: Survey of Techniques and a Novel Omni-Video-Based Approach for Dynamic Panoramic Surround Maps. IEEE Trans. Intell. Transp. Syst..

[12] Srijongkon K., Duangsoithong R., Jindapetch N., Ikura M., Chumpol S. SDSoC based development of vehicle counting system using adaptive background method. Proceedings of the 2017 IEEE Regional Symposium on Micro and Nanoelectronics (RSM).

[13] Prommool P., Auephanwiriyakul S., Theera-Umpon N. Vision-based automatic vehicle counting system using motion estimation with Taylor series approximation. Proceedings of the 2016 6th IEEE International Conference on Control System, Computing and Engineering (ICCSCE).

[14] Swamy G.N., Srilekha S. Vehicle detection and counting based on color space model. Proceedings of the 2015 International Conference on Communications and Signal Processing (ICCSP).

[15] Seenouvong N., Watchareeruetai U., Nuthong C., Khongsomboon K., Ohnishi N. A computer vision based vehicle detection and counting system. Proceedings of the 2016 8th International Conference on Knowledge and Smart Technology (KST).

[16] Ke R., Kim S., Li Z., Wang Y. Motion-vector clustering for traffic speed detection from UAV video. Proceedings of the 2015 IEEE First International Smart Cities Conference (ISC2).

[17] Shastry A.C., Schowengerdt R.A. (2005). Airborne video registration and traffic-flow parameter estimation. IEEE Trans. Intell. Transp. Syst..

[18] Cao X., Gao C., Lan J., Yuan Y., Yan P. (2014). Ego Motion Guided Particle Filter for Vehicle Tracking in Airborne Videos. Neurocomput.

[19] Pouzet M., Bonnin P., Laneurit J., Tessier C. Moving targets detection from UAV based on a robust real-time image registration algorithm. Proceedings of the 2014 IEEE International Conference on Image Processing (ICIP).

[20] Freis S., Olivares-Mendez M.A., Viti F. Estimating speed profiles from aerial vision—A comparison of regression based sampling techniques. Proceedings of the 2016 24th Mediterranean Conference on Control and Automation (MED).

[21] Chen X., Meng Q. Vehicle Detection from UAVs by Using SIFT with Implicit Shape Model. Proceedings of the 2013 IEEE International Conference on Systems, Man, and Cybernetics.

[22] Guvenc I., Koohifar F., Singh S., Sichitiu M.L., Matolak D. (2018). Detection, Tracking, and Interdiction for Amateur Drones. IEEE Commun. Mag..

[23] Shi X., Ling H., Blasch E., Hu W. Context-driven moving vehicle detection in wide area motion imagery. Proceedings of the 21st International Conference on Pattern Recognition (ICPR2012).

[24] LaLonde R., Zhang D., Shah M. ClusterNet: Detecting Small Objects in Large Scenes by Exploiting Spatio-Temporal Information. Proceedings of the IEEE Conference on Computer Vision and Pattern Recognition (CVPR).

[25] Wang P., Yan X., Gao Z. Vehicle counting and traffic flow analysis with UAV by low rank representation. Proceedings of the 2017 IEEE International Conference on Robotics and Biomimetics (ROBIO).

[26] Barnich O., Droogenbroeck M.V. (2011). ViBe: A Universal Background Subtraction Algorithm for Video Sequences. IEEE Trans. Image Process..

[27] Bay H., Ess A., Tuytelaars T., Gool L.V. (2008). Speeded-Up Robust Features (SURF). Comput. Vis. Image Understand..

[28] Fischler M.A., Bolles R.C. (1981). Random Sample Consensus: A Paradigm for Model Fitting with Applications to Image Analysis and Automated Cartography. Commun. ACM.

[29] Kalal Z., Mikolajczyk K., Matas J. (2012). Tracking-Learning-Detection. IEEE Trans. Pattern Anal. Mach. Intell..

[30] Hare S., Golodetz S., Saffari A., Vineet V., Cheng M.M., Hicks S.L., Torr P.H.S. (2016). Struck: Structured Output Tracking with Kernels. IEEE Trans.Pattern Anal. Mach. Intell..

[31] Henriques J.F., Caseiro R., Martins P., Batista J. (2015). High-Speed Tracking with Kernelized Correlation Filters. IEEE Trans.Pattern Anal. Mach. Intell..

[32] Dalal N., Triggs B. Histograms of oriented gradients for human detection. Proceedings of the 2005 IEEE Computer Society Conference on Computer Vision and Pattern Recognition (CVPR’05).

